# Vitamin D Supplementation for 12 Months in Older Adults Alters Regulators of Bone Metabolism but Does Not Change Wnt Signaling Pathway Markers

**DOI:** 10.1002/jbm4.10619

**Published:** 2022-03-24

**Authors:** Marilena Christodoulou, Terence J Aspray, Isabelle Piec, Christopher Washbourne, Jonathan CY Tang, William D Fraser, Inez Schoenmakers, the VDOP Trial Group

**Affiliations:** ^1^ Medical School University of East Anglia Norwich UK; ^2^ Freeman Hospital, Bone Clinic University of Newcastle upon Tyne Newcastle upon Tyne UK

**Keywords:** BONE TURNOVER, FIBROBLAST GROWTH FACTOR‐23, PARATHYROID HORMONE, VITAMIN D, WNT PATHWAY

## Abstract

Vitamin D status and supplementation regulates bone metabolism and may modulate Wnt signaling. We studied the response of hormonal regulators of bone metabolism, markers of Wnt signaling and bone turnover and bone mineral density (BMD) and bone mineral content (BMC) in a randomized vitamin D intervention trial (12,000 IU, 24,000 IU, 48,000 IU/mo for 1 year; men and women aged >70 years; *n* = 379; ISRCTN35648481). Associations with total and free 25(OH)D concentrations were analyzed by linear regression. Baseline vitamin D status was (mean ± SD) 25(OH)D: 40.0 ± 20.1 nmol/L. Supplementation dose‐dependently increased total and free 25(OH)D concentrations and decreased plasma phosphate and parathyroid hormone (PTH) (all *p* < 0.05). The procollagen 1 intact N‐terminal (PINP)/C‐terminal telopeptide (CTX) ratio, C‐terminal fibroblast growth factor‐23 (cFGF23), and intact FGF23 (iFGF23) significantly increased with no between‐group differences, whereas Klotho was unchanged. 1,25(OH)_2_D and PINP significantly increased in the 24 IU and 48,000 IU groups. Sclerostin (SOST), osteoprotegerin (OPG), receptor activator of NF‐κB ligand (RANKL), BMD, BMC, and CTX remained unchanged. Subgroup analyses with baseline 25(OH)D <25 nmol/L (*n* = 94) provided similar results. Baseline total and free 25(OH)D concentrations were positively associated with 1,25(OH)_2_D, 24,25(OH)_2_D (*p* < 0.001), vitamin D binding protein (DBP) (*p* < 0.05), BMD, and BMC (*p* < 0.05). Associations with PTH (*p* <0.001), cFGF23 (*p* < 0.01), and BAP (*p* < 0.05) were negative. After supplementation, total and free 25(OH)D concentrations remained positively associated only with 24,25(OH)_2_D (*p* < 0.001) and DBP (*p* < 0.001) and negatively with estimated glomerular filtration rate (eGFR) (*p* < 0.01). PTH and SOST were significantly associated only with free 25(OH)D. There were no significant relationships with BMD and BMC after supplementation. The decrease in PTH and increase in PINP/CTX ratio suggest a protective effect of supplementation on bone metabolism, although no significant effect on BMD or pronounced changes in regulators of Wnt signaling were found. The increase in FGF23 warrants caution because of its negative association with skeletal and cardiovascular health. Associations of total and free 25(OH)D with biomarkers were similar and known positive associations between vitamin D status and BMD were confirmed. The change in associations after supplementation might suggest a threshold effect. © 2022 The Authors. *JBMR Plus* published by Wiley Periodicals LLC on behalf of American Society for Bone and Mineral Research.

## Introduction

There is limited evidence to suggest that 25 hydroxy vitamin D (25(OH)D), 1,25 di‐hydroxy vitamin D (1,25(OH)_2_D), and vitamin D analogue supplementation influence the regulatory function of the osteocyte.^(^ ^1^ ^)^ The mechanisms are only partly understood.^(^ ^2^ ^)^ The Wnt/β‐catenin pathway plays a key role in bone remodeling.^(^ ^3^ ^)^ The effect of Wnt/β‐catenin signaling is mainly anabolic^(^ ^4^ ^)^ as it increases osteoblast differentiation and osteocyte function^(^ ^3^ ^)^ and inhibits osteoclasts differentiation. This occurs through upregulation of osteoprotegerin (OPG),^5^, ^6^ ^)^ which binds to the receptor activator of NF‐κB ligand (RANKL), preventing interaction with its receptor, RANK.^(^ ^7^ ^)^ This mechanism is regulated by a multitude of factors, for example, transforming growth factor beta (TGF‐β), sclerostin (SOST), and Dickkopf‐related protein 1 (DKK1).^(^ ^7^ ^)^ Both SOST and DKK1 act as inhibitors of the Wnt/β‐catenin pathway, causing bone loss,^(^ ^3^ ^)^ although in cross‐sectional studies positive associations between plasma SOST and BMD were reported.^(^ ^8^, ^9^, ^10^ ^)^ The production of SOST is regulated by a variety of factors such as mechanical loading, TGF‐β, and parathyroid hormone (PTH).^(^ ^3^, ^11^, ^12^, ^13^ ^)^ Also, Klotho and FGF23 influence bone mineralization, and both low and elevated concentrations of Klotho and FGF23 impair this,^(^ ^14^, ^15^ ^)^ partly through their effects on vitamin D metabolism^(^ ^16^ ^)^ and calcium and phosphate homeostasis.^(^ ^17^, ^18^, ^19^ ^)^

Aging is associated with changes in the renal‐bone axis and increased resistance to the regulating hormones (PTH, 1,25(OH)_2_D, and FGF23) and renal impairment.^(^ ^20^, ^21^, ^22^ ^)^ Plasma concentrations of SOST and FGF23 increase with age and are elevated from the early stages of renal impairment, whereas the FGF23 receptor co‐factor, αKlotho, declines.^(^ ^8^ ^)^ These changes may lead to a reduction in Wnt signaling and eventually to loss of bone mass and integrity.^(^ ^20^ ^)^ The latter may be particularly detectable in trabecular bone, the partition that is the most metabolically active and may not be detected by dual‐energy X‐ray absorptiometry (DXA).^(^ ^8^, ^10^ ^)^ Because these changes may be subject to long‐term programming,^(^ ^23^ ^)^ prevention is critical.

Vitamin D deficiency (here defined as 25(OH)D concentrations <25 nmol) is associated with secondary hyperparathyroidism and contributes to alterations in bone metabolism and bone loss.^(^ ^24^ ^)^ Optimizing vitamin D status may therefore be of particular importance in the older population. Although the effect of vitamin D supplementation, without concomitant calcium supplementation, on loss of bone mineral density (BMD) and bone mineral content (BMC) is small, the effects on the regulation of bone metabolism through Wnt signaling are poorly characterized.^(^ ^25^ ^)^ Limited research studies suggest that SOST decreases with vitamin D supplementation.^(^ ^26^, ^27^ ^)^

Randomized controlled trials (RCT) of vitamin D supplementation with musculoskeletal outcomes have shown the importance of considering the relationship between plasma concentrations of 25(OH)D and outcomes.^(^ ^25^ ^)^ This is because there is considerable between‐individual variation in the dose‐response to supplementation and trials may be confounded by baseline status and vitamin D supply from other sources (dietary intake and cutaneous synthesis).

This study aimed to investigate changes in regulators and markers of bone metabolism, BMD, and BMC in response to different dosages of vitamin D supplementation in older people for 12 months. We investigated four categories of markers: (i) calcium metabolism and renal function, (ii) vitamin D metabolites, (iii) Wnt signaling, and (iv) bone parameters and bone metabolism. Further, we investigated their associations with total 25(OH)D and free 25(OH)D at baseline and after 12 months of supplementation.

## Materials and Methods

### Study design

This study is a secondary analysis utilizing plasma samples collected as part of a dose‐ranging randomized vitamin D intervention trial in older people (VDOP)^(^ ^28^ ^)^ (ISRCTN35648481 and EudraCT 2011–004890‐10). In brief, this RCT included 379 adults aged ≥70 years (48% women; mean age 75 years) from the northeast of England. Participants were randomly allocated to 1 of 3 doses of vitamin D_3_ (12,000 international units [IU], 24,000 IU, or 48,000 IU) given once a month for a year. The 12,000 IU and 24,000 IU dosages correspond to the UK Dietary Reference Nutrient Intake (RNI) of 400 IU/d (10 μg/d)^(^ ^24^ ^)^ and the North American Recommended Dietary Allowance (RDA) of 800 IU/d (20 μg/d) for people over 70 years old.

This study was powered to detect a change in BMD at the hip in response to 12,000, 24,000, or 48,000 IU vitamin D_3_/m for 1 year, using 12,000 IU as the reference dose. The power calculation was based on findings in an earlier, similar study in the North of the UK.^(^ ^28^, ^29^ ^)^ Detailed description of the design, methods, and primary outcomes of VDOP were earlier published.^(^ ^28^, ^30^ ^)^ Results for bone mineral density and bone area (at hip and femoral neck), plasma concentrations of 25(OH)D, PTH, albumin, calcium, and creatinine were earlier reported but are also included here as part of secondary analyses and to support data interpretation.

Additional methods used for these secondary analyses are provided below. These explorative secondary analyses were not prespecified in the original trial design and analyses plan.

The study was conducted in accordance with guidelines laid down in the Declaration of Helsinki. A favorable opinion was obtained from the Tyne & Wear South Research Ethics Committee (REC, 12/NE/0050) with Research and Development approval from the sponsor, Newcastle upon Tyne Hospitals NHS Foundation Trust. All participants provided written informed consent.

### Measurements

Measurements of BMD and BMC at the hip and femoral neck (FN), height, and weight were taken.^(^ ^28^ ^)^ Early morning fasting blood samples were collected from all participants at baseline and after 12 months of supplementation. Plasma calcium, albumin, and creatinine were measured by Newcastle upon Tyne Hospitals NHS Foundation Trust (NUTH) laboratories and the analysis was carried out immediately after sample collection.

The remaining blood samples were placed on ice and separated within 30 minutes of collection in a refrigerated centrifuge at 1800*g* for 20 minutes. Plasma was transported on dry ice and stored at −80°C. Biochemical analysis took place at Medical Research Council (MRC) Human Nutrition Research, Cambridge, UK. The assay specifications were as described before.^(^ ^28^ ^)^ Analyses specific for this secondary study were conducted at Bioanalytical Facility of University of East Anglia (UEA), UK, and are specified below.

In brief, analyses included 25(OH)D (LC–MS/MS), vitamin D binding protein (DBP) (Immundiagnostik AG, Bensheim, Germany; ELISA), PTH (Immulite 2000, Siemens, Munich, Germany), procollagen 1 intact N‐terminal (PINP) (UniQ RIA, Aidian Oy, Espoo, Finland), C‐terminal telopeptide (CTX) (Immunodiagnostic Systems, Boldon, UK), and bone‐specific alkaline phosphatase (BAP) (LIAISON, DiaSorin, Stillwater, MN, USA). All assays were performed in duplicate except for PTH. Assay performance was monitored using kit and in‐house controls and under strict standardization according to ISO 9001:2000. External quality assurance of 25(OH)D and PTH assays were performed as part of the Vitamin D External Quality Assessment Scheme (www.deqas.org) and the National External Quality Assessment Scheme (www.ukneqas.org.uk). Measurements of 25(OH)D were harmonized against NIST standards as part of the Vitamin D harmonization program.^(^ ^28^ ^)^

Measurements conducted at UEA included serum phosphate (Phosphate (Inorganic) ver.2, Cobas, Roche, Mannheim, Germany), αKlotho (IBL International, Hamburg, Germany), C‐terminal fibroblast growth factor‐23 (cFGF23; Gen 2, Immutopics, San Clemente, CA, USA), intact FGF23 (iFGF23; Gen 2, Immutopics), OPG (Biomedica, Vienna, Austria), SOST (Biomedica), DKK1 (Biomedica), soluble RANKL (sRANKL, Biomedica), 24,25(OH)_2_D (LC–MS/MS),^(^ ^31^ ^)^ 1,25(OH)_2_D (Diasorin, Liaison XL assay), and Cystatin C (Tina‐quant Cystatin C Gen 2). All assays were performed in duplicate except for 1,25(OH)_2_D, Cystatin C and phosphate. The inter‐ and intra‐assay coefficient of variation (CV) of all assays were <10% except for 24,25(OH)_2_D and 1,25(OH)_2_D, which were <15%. Assay performance was monitored using kit and in‐house controls and following Good Laboratory Practice.

### Derived variables

The estimated glomerular filtration rate (eGFR) was calculated using the Modification of Diet in Renal Disease (MDRD‐4) algorithm.

Calculated ratios included 25(OH)D/24,25(OH)_2_D, 1,25(OH)_2_D/24,25(OH)_2_D, PINP/CTX, sRANKL/OPG, and cFGF23/iFGF23 and were expressed as on molar/molar ratio, except for PINP/CTX and cFGF23/iFGF23.

Free 25(OH)D was calculated using the equation^(^ ^32^ ^)^:

$$\text{Free}\;25\left(\mathrm{OH}\right)D=\text{total}\;25\left(\mathrm{OH}\right)D/[1+\left(6*10^{3}\times \text{Albumin}\right)+\left(7*10^{8}\times \mathrm{DBP}\right)$$

### Statistical analysis

The findings presented in this article are the results of explorative secondary analyses. The primary outcome of the VDOP study was the change in BMD at the hip. A formal power calculation for secondary outcomes was not conducted but instead an estimation of the detectable effect size is provided for SOST data. In addition, correction for repeated testing was not deemed appropriate for this explorative analysis as any finding will require confirmation in RCTs specifically designed and powered for respective outcomes.

The sample size calculation was based on a detectable effect size (a 15% reduction in plasma SOST in any arm of study). This is within the observed % reduction of SOST after treatment with pharmaceutical agents to reduce bone resorption. Data from a study in older men and women (>65 years of age; *n* = 95) provided an estimate of the biological variability of SOST.^(^ ^33^ ^)^ The mean (SD) plasma SOST concentration was 27.8 (14.1) ng/mL. The between‐subject CV% [(CV); SD*100/mean %)] was 51%. Other data suggest a CV of 30% to 42%.^(^ ^34^, ^35^, ^36^ ^)^ It is assumed that the within‐subject variation is approximately half the size of the between‐subject estimate, that is 25% to 30%. The sample size calculation was based on a conservative CV% of 30%. To detect an effect size of 15%, with a 30% CV, 5% significance level, and 90% power, the required sample size is 84 subjects per arm. Samples available from the VDOP study were *n* = 113, 114, and 116/arm.

Before *t* tests and ANCOVA analyses, all outcomes were assessed for normality (defined as a posterior distribution skewness <2 or >−2) and visual inspection of histograms. Non‐normally distributed variables were converted to natural logarithm values (LN) and checked again for normality. The distributions of Klotho and cFGF23 at both time points (baseline and 12 months) were extremely skewed. Outliers were identified on basis of *Z*‐scores (based on interquartile range [IQR]) and excluded if <−2.68 or >2.68. After excluding the extreme outliers, the LN values of both variables were normally distributed. Analyses were conducted with and without these outliers and there were no material differences between outcomes and interpretation of the data.

Differences between pre‐ and post‐supplementation values were tested with paired sample *t* tests for each supplementation group. Between‐group differences post‐supplementation were tested by ANCOVA, with the baseline value as covariate. Additional models included eGFR and sex as covariates. These models did not provide substantially different results and/or these covariates were nonsignificant and therefore only the result of the ANCOVAs with the baseline value as covariate were reported, unless stated otherwise. Data were presented as mean and SD or median and IQR for normally distributed and skewed data, respectively.

To assess whether vitamin D deficiency at baseline influenced the effect of supplementation, analyses as described above were conducted separately for participants with a plasma 25(OH)D ≤25 nmol/L at baseline.

Regression analysis was used to test associations with total and free 25(OH)D concentration before and after 12 months of supplementation. For post‐supplementation data, the dose was entered as a covariate but was nonsignificant. Therefore, results of univariate models are presented. Regression analyses for variables derived from the independent variable were not conducted (ie, for free 25(OH)D, these were DBP, Alb, and total 25(OH)D) and for any of the ratios with total 25(OH)D). Linearity of associations was visually inspected. Two outliers for free 25(OH)D were excluded from the 12‐month data. Results are presented as the β‐coefficient and associated *p* value.

For the statistical analysis of the data, IBM (Armonk, NY, USA) SPSS Statistics version 25 software was used.

## Results

Baseline characteristics are presented in Table 1. Baseline characteristics were well balanced between treatment groups and no significant differences were found.

**Table 1:** Participants' Characteristics and Response to Vitamin D Supplementation^a^

*n*	Baseline	12 months (12,000 IU)	12 months (24,000 IU)	12 months (48,000 IU)	ANCOVA^b^ analysis
	379	122	124	126	
Age (years)	74.1 [71.5–77.0]	75.6 [72.5–77.3]	76.0 [72.5–77.9]	76.4 (4.4)	n/a
Plasma calcium and renal function markers					
Albumin (g/L)	45.7 (2.2)	44.6 (2.1)*	44.5 (2.6)*	44.3 (2.0)*	0.70
Adjusted calcium (mmol/L)	2.2 (0.1)	2.3 (0.1)	2.2 (0.1)	2.2 (0.1)	0.07
Phosphate (mmol/L)	0.88 (0.18)	0.79 (0.19)*	0.81 (0.17)*	0.81 (0.19)*	0.68
Cystatin C (mg/L)	0.87 (0.22)	0.88 (0.27)	0.92 (0.25)*	0.95 (0.29)*	0.41
Creatinine (μmol/L)	82.1 (19.1)	82.5 [67–94]*	84.6 [71–96]*	78.1 (66–88]*	0.25
eGFR (mL/min per 1.73 m^2^)	72 (15)	73 (14)	74 (16)	70 (15)	0.54
Klotho (pg/mL)	493.7 [392.6–627.7]	502.0 [403.1–639.6]	502.7 [395.5–611.2]	477.6 [399.6–589.2]	<0.001
cFGF23 (RU/mL)	66.7 [54.9–84.2]	90.8 [65.3–104.9]*	85.7 [59.7–94.9]*	77.8 [58.4–87.3]*	0.11
iFGF23 (pg/mL)	55.1 [44.5–72.7]	66.9 [42.5–88.4]*	71.7 [54.5–79.4]*	73.2 [52.2–84.7]*	0.80
iFGF23/cFGF23	1.9 [1.5–2.5]	2.1 [1.4–2.2]	2.2 [1.4–2.3]	2.3 [1.4–2.3]	0.75
Vitamin D metabolism markers					
Total 25(OH)D (nmol/L)	40.0 (20.1)	55.9 (15.6)*	64.6 (15.3)*	79.0 (15.1)*	<0.001
Free 25(OH)D (pmol/L)	8.4 (4.3)	11.7 (3.3)*	13.8 (3.4)*	16.9 (4.3)*	<0.001
24,25(OH)_2_D (nmol/L)	3.2 [2.0–5.5]	6.1 (2.7)*	7.4 (2.8)*	9.4 (3.0)*	<0.001
25(OH)D/24,25(OH)_2_D	14.5 [11.3–18.8]	12.1 [9.4–12.9]*	11.9 [10.0–13.4]*	12.9 (4.7)*	<0.001
1,25(OH)_2_D (pmol/L)	94.5 (29.0)	100.6 (29.8)	101.0 (29.4)*	101.9 (30.8)*	0.099
1,25(OH)_2_D/24,25(OH)_2_D	25.7 [16.8–44.4]	17.7 [11.9–21.8]	14.6 [8.9–18.7]	15.3 [9.0–18.2]	<0.001
DBP (mg/L)	367.8 (63.4)	362.5 (74.1)	356.9 (46.1)	384.4 (57.8)*	<0.01
PTH (pg/mL)	43.4 [33.2–57.4]	39.8 [28.8–53.5]*	40.9 [26.3–55.5]*	37.3 [27.8–47.5]*	<0.01
Wnt signaling pathway markers					
SOST (pmol/L)	44.3 [32.4–60.0]	46.9 [32.6–63.5]	45.4 [32.1–57.8]	46.5 [33.2–61.2]	0.20
DKK1 (pmol/L)	31.2 (16.5)	40.6 (17.9)*	33.2 (19.0)	38.9 (18.1)*	0.87
OPG (pmol/L)	5.67 (2.08)	5.69 (2.04)	5.12 [4.25–6.47]	5.89 (2.17)	0.20
sRANKL (pmol/L)	0.12 [0.08–0.18]	0.14 [0.08–0.18]	0.13 [0.07–0.18]	0.14 (0.07)	0.75
sRANKL/OPG	0.02 [0.01–0.04]	0.03 [0.01–0.03]	0.04 [0.01–0.04]	0.03 [0.01–0.04]	0.85
Bone mineral density and metabolism					
Hip BMD (g/m^2^)	0.98 (0.17)	0.96 (0.15)	0.98 (0.16)	0.99 (0.18)	0.19
Hip BMC (g)	35.44 (8.30)	34.08 (7.56)	35.42 (7.92)	35.73 (8.63)	0.14
FN BMD (g/m^2^)	0.902 (0.152)	0.88 (0.13)	0.90 (0.14)	0.92 (0.15)	0.13
FN BMC (g)	4.90 (1.09)	4.78 (0.96)	4.82 (1.04)	4.70 (1.16)	0.72
BAP (μg/L)	9.5 [7.9–12.3]	11.4 [8.4–13.8]*	10.7 [7.7–12.7]	11.4 [8.0–14.1]	0.87
CTX (ng/mL)	0.40 [0.30–0.50]	0.36 (0.16)	0.37 (0.15)	0.35 (0.14)	0.48
PINP (μg/L)	36.2 [28.8–46.2]	40.1 [31.7–52.6]	39.1 [31.0–46.6]*	38.4 [28.9–47.1]*	0.53
PINP/CTX	101.4 [85.9–116.9]	120.5 [103.6–155.2]*	124.0 [107.6–158.5]*	118.8 [100.8–157.1]*	0.99

1,25(OH)_2_D = 1,25‐dihydroxy vitamin D; 24,25(OH)_2_D = 24,25‐dihydroxy vitamin D; 25(OH)D = 25‐hydroxy vitamin D; BAP = bone‐specific alkaline phosphatase; BMC = bone mineral content; BMD = bone mineral density; cFGF23 and iFGF23 = c‐terminal and intact fibroblast growth factor‐23, respectively; CTX = C‐terminal telopeptide; DBP = vitamin D binding protein; DKK1 = Dickkopf‐related protein 1; eGFR = estimated glomerular filtration rate; FN = femoral neck; OPG = osteoprotegerin; PINP = procollagen 1 intact N‐terminal; PTH = parathyroid hormone; SOST = sclerostin; sRANKL = soluble receptor activator of NF‐κB ligand.

^a^ For normally distributed data, results are expressed as mean (SD); skewed results are expressed as median [interquartile range].

^b^ ANCOVA was used to test between‐group differences after 12 months of supplementation, with the baseline value as a covariate.

*Denotes significantly different from baseline *p* < 0.05. Paired *t* tests were used to analyze pre‐ and post‐supplementation values for each supplementation group.

### Plasma calcium and renal function markers

Adjusted calcium remained unaltered. There was a significant decrease in plasma phosphate in all treatment groups compared with baseline (*p* < 0.01), but there was no dose effect. There were significant changes in plasma creatinine in all treatment groups (*p* < 0.001), with no between‐group differences but eGFR was unaltered. Plasma cFGF23 and iFGF23 significantly increased in all treatment groups with supplementation (*p* < 0.05) without a significant dose effect. The cFGF23/iFGF23 ratio remained unaltered (Table 1). Although Klotho remained unaltered compared with baseline, there were significant between‐group differences (*p* < 0.001) after supplementation.

In ANCOVA models for albumin and cFGF23, eGFR was a significant covariate. Inclusion of eGFR in these models did not alter the interpretation of findings.

### Vitamin D metabolism

Post‐supplementation, all vitamin D metabolites were significantly higher in all treatment groups (*p* < 0.001) compared with baseline, except 1,25(OH)_2_D, which only significantly increased in the 24,000 IU and 48,000 IU groups (both *p* < 0.01). Supplementation had a significant dose‐dependent effect on total, free 25(OH)D, and 24,25(OH)_2_D (all *p* < 0.001). DBP was unchanged, except for a significant increase in the 48,000 IU group (*p* < 0.05). PTH decreased in all treatment groups after supplementation (*p* < 0.05) with a significant dose‐dependent effect (*p* < 0.001) (Table 1).

In ANCOVA models for total and free 25(OH)D, eGFR was a significant covariate. Both models with and without this covariate were significant (both *p* < 0.001).

### Wnt signaling pathway markers

There were no changes with supplementation in plasma concentrations of SOST, OPG, sRANKL, and sRANKL/OPG ratio. Differences between groups were nonsignificant. DKK1 significantly increased in the 12,000 IU and 48,000 IU groups (*p* < 0.05) and there were significant differences between the treatment groups (*p* < 0.05) (Table 1).

### Bone parameters and markers of bone metabolism

BMD and BMC at the hip, FN, and CTX were not significantly different compared with baseline, and there were no group differences as reported before.^(^ ^28^, ^30^ ^)^ Femoral neck area was significantly lower in the 48,000 IU group, and there were significant differences between the groups after supplementation (*p* < 0.001). BAP significantly increased only in the 12,000 IU group (*p* < 0.05). PINP/CTX ratio significantly increased, respectively, with supplementation in all treatment groups (all *p* < 0.001). PINP significantly increased compared with baseline only in the 24,000 IU and 48,000 IU groups (*p* < 0.001) with no differences between groups (Table 1).

### Subgroup analyses in participants vitamin D deficient at baseline

At baseline, 28% of participants had a 25(OH)D concentration ≤25 nmol/L (mean 25(OH)D: 18.8 ± 4.1 nmol/L), and the numbers were equally distributed between supplementation groups.^(^ ^27^ ^)^ This group had a significantly lower hip and FN BMD and BMC. Plasma concentrations of 1,25(OH)_2_D, 24,25(OH)_2_D, and PINP were lower and PTH and cFGF23 were higher compared with the group with baseline 25(OH)D >25 nmol/L (all *p* < 0.05).

In line with the findings in the full cohort, with supplementation, no significant changes in hip and FN BMD and BMC were found. Supplementation significantly increased concentrations of total and free 25(OH)D, 24,25(OH)_2_D, and cFGF23 concentrations and decreased PTH in all supplementation groups. Plasma iFGF23 significantly increased with the two highest dosages. Plasma 1,25(OH)_2_D was significantly higher in all treatment groups. Klotho, SOST, OPG, RANKL, BAP, and CTX remained unchanged. The observed increase in PINP in the full cohort was not found but instead a PINP significantly decreased in the 24,000 IU group but the PINP/CTX ratio significantly increased in all groups. The observed decrease in plasma phosphate was not found in this subgroup.

### Associations with total and free 25(OH)D plasma concentrations

#### Plasma calcium and renal function markers

**Fig. 1:**
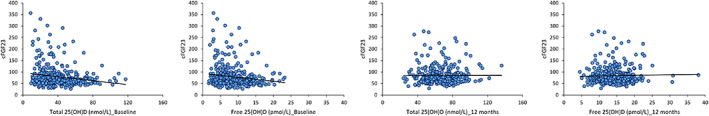
Correlations of total and free 25(OH)D with cFGF23 at baseline and 12 months.

At baseline, total and free 25(OH)D were significantly negatively associated with cFGF23 (*p* < 0.001) (Fig. 1) and the cFGF23/iFGF23 ratio (*p* < 0.05) (Table 2).

**Table 2:** Associations of Total and Free 25(OH)D With Biomarkers at Baseline and 12 Months

	Baseline				12 months			
	Total 25(OH)D (nmol/L)		Free 25(OH)D (pmol/L)		Total 25(OH)D (nmol/L)		Free 25(OH)D (pmol/L)	
	β coefficient	*p* value	β coefficient	*p* value	β coefficient	*p* value	β coefficient	*p* value
Plasma calcium and renal function markers								
Albumin (g/L)	0.004	0.44	n/a	n/a	−0.003	0.72	n/a	n/a
Adjusted calcium (mmol/L)	0.000	0.34	0.001	0.46	**−0.001**	**<0.05**	**−0.002**	**<0.05**
Serum phosphate (mmol/L)	0.001	0.08	0.003	0.18	0.000	0.84	0.003	0.18
Cystatin C (mg/L)	0.000	0.50	−0.002	0.425	0.000	0.88	0.004	0.19
Serum creatinine (μmol/L)	0.027	0.58	0.169	0.47	−0.074	0.23	−0.424	0.10
eGFR (mL/min per 1.73 m^2^)	0.026	0.50	0.144	0.43	**−0.141**	**<0.01**	**−0.710**	**<0.001**
Klotho (pg/mL)	−1.398	0.40	−7.066	0.37	−1.606	0.10	−1.771	0.66
cFGF23 (RU/mL)	**−0.433**	**<0.001**	**−1.741**	**<0.01**	−0.203	0.10	−0.396	0.45
iFGF23 (pg/mL)	0.107	0.22	0.227	0.50	0.047	0.68	0.485	0.31
iFGF23/cFGF23	**−0.023**	**<0.05**	**−0.104**	**<0.05**	−0.005	0.21	−0.006	0.74
Vitamin D metabolism markers								
24,25(OH)_2_D (nmol/L)	**0.126**	**<0.001**	**0.559**	**<0.001**	**0.124**	**<0.001**	**0.452**	**<0.001**
1,25(OH)_2_D (pmol/L)	**0.425**	**<0.001**	**1.964**	**<0.001**	0.130	0.15	0.268	0.49
1,25(OH)_2_D:24,25(OH)_2_D	**−0.916**	**<0.001**	**−0.114**	**<0.001**	−0.010	0.73	−0.015	0.91
DBP (mg/L)	**0.439**	**<0.01**	n/a	n/a	**0.803**	**<0.001**	n/a	n/a
PTH (pg/mL)	**−0.360**	**<0.001**	**−1.612**	**<0.001**	−0.121	0.09	**−0.752**	**<0.05**
Wnt signaling pathway markers								
SOST (pmol/L)	0.020	0.76	0.237	0.45	0.014	0.86	**0.634**	**<0.05**
DKK1 (pmol/L)	0.000	0.99	−0.03	0.89	0.035	0.55	0.194	0.46
OPG (pmol/L)	0.002	0.76	−0.016	0.53	0.005	0.51	0.024	0.45
sRANKL (pmol/L)	0.000	0.90	0.000	0.79	0.000	0.78	0.000	0.66
sRANKL/OPG	0.000	0.88	0.000	0.82	0.000	0.85	0.000	0.94
Bone density and metabolism								
Hip BMD (g/m^2^)	**0.001**	**<0.01**	**0.006**	**<0.01**	0.001	0.10	0.003	0.14
Hip BMC (g)	**0.053**	**<0.05**	**0.298**	**<0.01**	0.036	0.16	0.121	0.27
FN BMD (g/m^2^)	**0.001**	**<0.05**	**0.005**	**<0.01**	0.001	0.14	0.003	0.11
FN BMC (g)	**0.007**	**<0.05**	**0.039**	**<0.01**	0.000	0.99	0.000	0.99
BAP (μg/L)	**−0.019**	**<0.05**	**−0.096**	**<0.05**	0.006	0.72	0.038	0.58
CTX (ng/mL)	0.000	0.61	−0.002	0.43	0.000	0.51	−0.001	0.73
PINP (μg/L)	−0.034	0.42	−0.202	0.32	−0.031	0.63	−0.014	0.96
PINP/CTX	0.007	0.93	−0.005	0.99	−0.137	0.26	−0.315	0.54

25(OH)_2_D = 1,25‐dihydroxy vitamin D; 24,25(OH)_2_D = 24,25‐dihydroxy vitamin D; 25(OH)D = 25‐hydroxy vitamin D; BAP = bone‐specific alkaline phosphatase; BMC = bone mineral content; BMD = bone mineral density; cFGF23 and iFGF23 = c‐terminal and intact fibroblast growth factor‐23, respectively; CTX = C‐terminal telopeptide; DBP = vitamin D binding protein; DKK1 = Dickkopf‐related protein 1; eGFR = estimated glomerular filtration rate; FN = femoral neck; OPG = osteoprotegerin; PINP = procollagen 1 intact N‐terminal; PTH = parathyroid hormone; SOST = sclerostin; sRANKL = soluble receptor activator of NF‐κB ligand. Univariate linear regression analysis; the table displays the β coefficients and the ANOVA *p* value for the β coefficient. Regression analyses for variables derived from the independent variable were not conducted (ie, for free 25(OH)D, these were DBP, Alb, and total 25(OH)D) and for any of the ratios with total 25(OH)D).

β‐coefficients and associated *p*‐values from univariate linear regression analysis. Significant associations *p*<0.05 are indicted in bold.

After supplementation, total and free 25(OH)D were negatively associated with adjusted calcium (*p* < 0.05) and eGFR (*p* < 0.01 and *p* < 0.001, respectively) (Table 2). No associations were found with the rest of the biomarkers after supplementation (Table 2).

#### Vitamin D metabolism

**Fig. 2:**
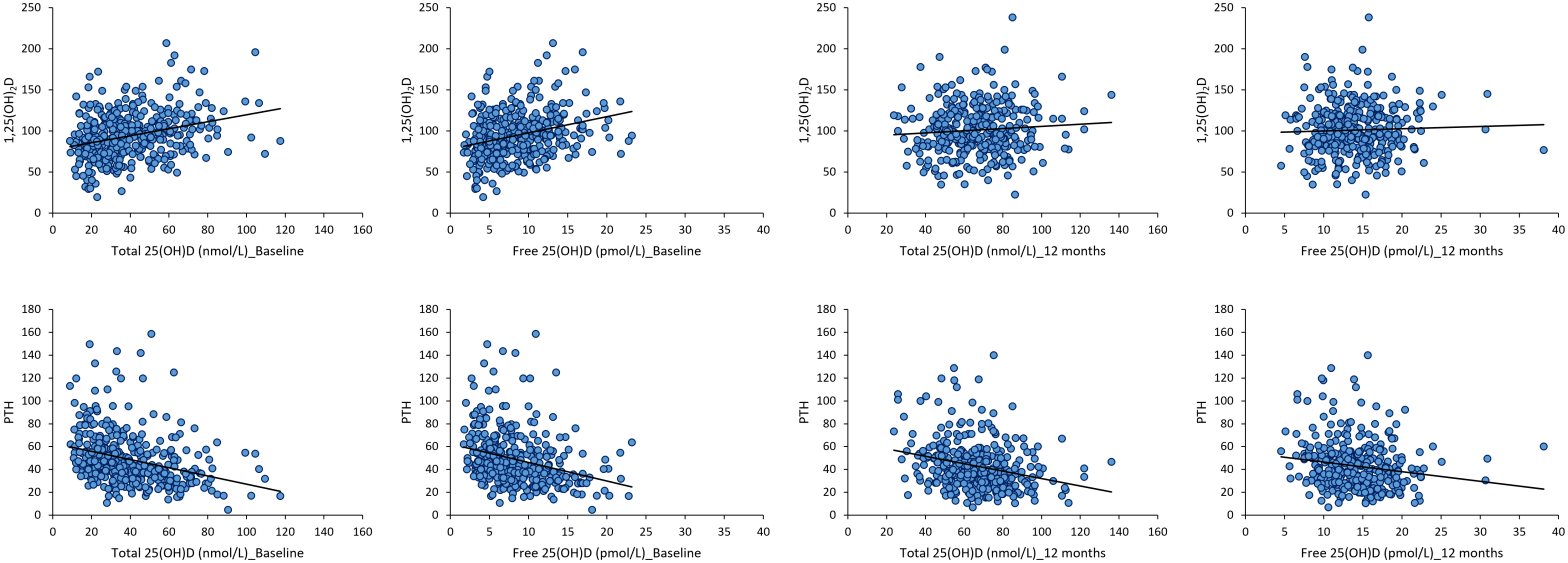
(*A*) Correlations of total and free 25(OH)D and 1,25(OH)_2_D at baseline and 12 months. (*B*) Correlations of total and free 25(OH)D and PTH at baseline and 12 months.

Pre‐supplementation, both total and free 25(OH)D were positively associated with 24,25(OH)_2_D and 1,25(OH)_2_D and negatively associated with 1,25(OH)2D/24,25(OH)_2_D and PTH (all *p* < 0.001) (Table 2; Fig. 2). DBP was positively associated with total 25(OH)D (*p* < 0.01) (Table 2).

Post‐supplementation, DBP and 24,25(OH)_2_D were positively associated with total 25(OH)D (both *p* < 0.001) (Table 2). Plasma PTH was negatively associated with free 25(OH)D (*p* < 0.05) and there was a tendency of significance for total 25(OH)D (*p* = 0.09)(Table 2; Fig. 2).

#### Wnt signaling pathway markers

**Fig. 3:**
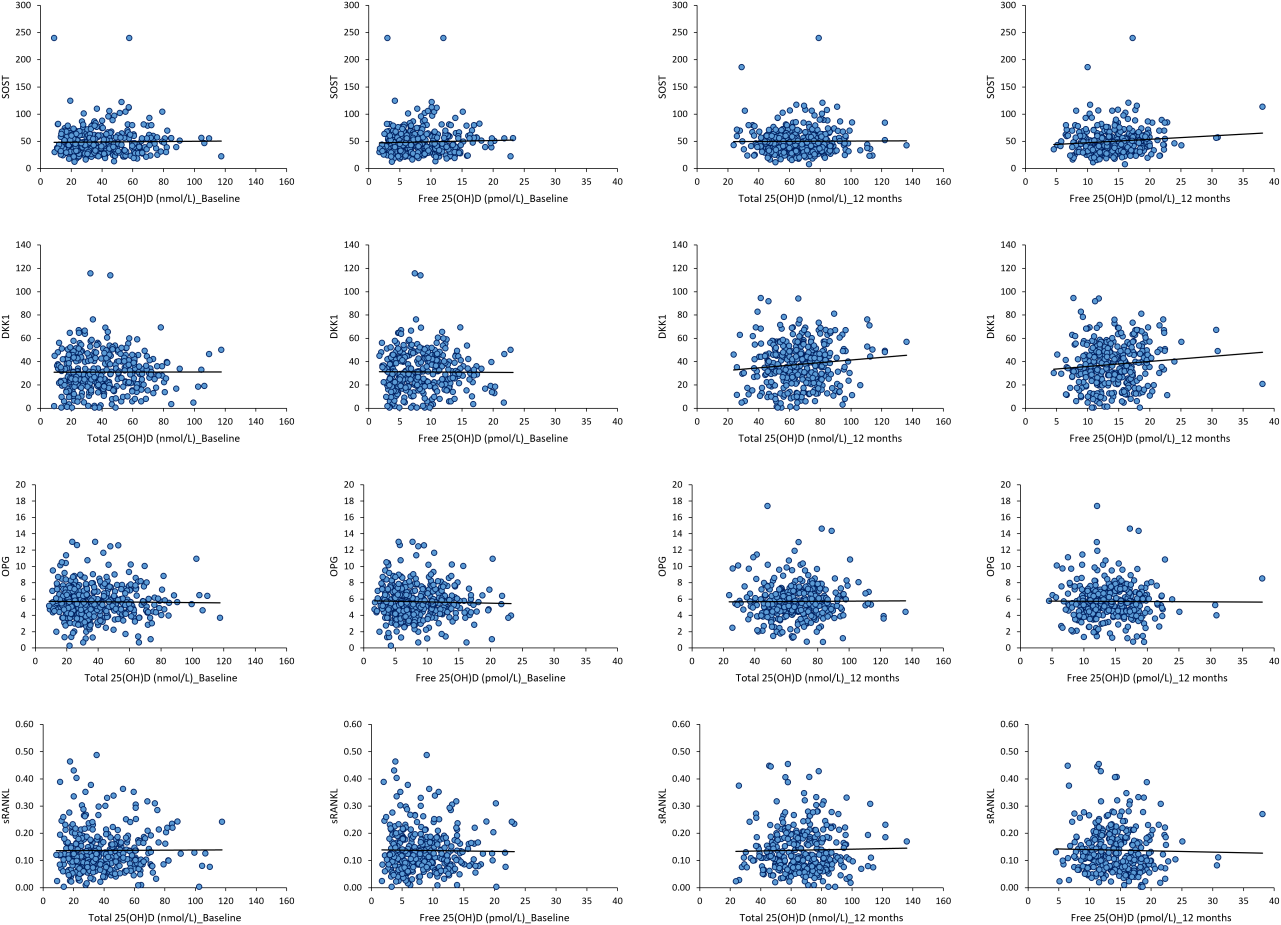
Correlations of 25(OH)D with Wnt signaling pathway markers at baseline and 12 months. (*A*) Correlations of total and free 25(OH)D and SOST. (*B*) Correlations of total and free 25(OH)D and DKK1. (*C*) Correlations of total and free 25(OH)D and OPG. (*D*) Correlations of total and free 25(OH)D and sRANKL.

The Wnt signaling markers DKK1, OPG, and sRANKL were not significantly associated with either total or free 25(OH)D both pre‐ and post‐supplementation. SOST was positively associated only with free 25(OH)D after supplementation (Table 2; Fig. 3).

#### Bone density and metabolism

**Fig. 4:**
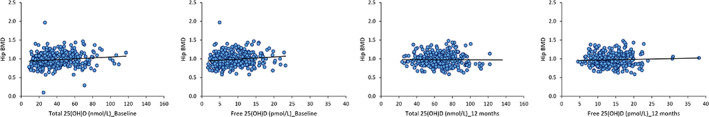
Correlations of total and free 25(OH)D with hip BMD at baseline and 12 months.

At baseline, both total and free 25(OH)D were positively associated with hip BMD (*p* < 0.01 and *p* <0.05, respectively), hip BMC (*p* < 0.05 and *p* < 0.01, respectively), FN BMC (*p* < 0.05 and *p* < 0.01, respectively) and FN BMD (both *p* < 0.05) (Table 2; Fig. 4). Of the bone metabolism markers, only BAP was significant (positively) associated with total and free 25(OH)D (*p* < 0.05) (Table 2).

After supplementation, no significant associations were found (Table 2).

## Discussion

Supplementation dose‐dependently increased total and free 25(OH)D concentrations and decreased plasma phosphate and PTH in all groups (all *p* < 0.05). The PINP/CTX ratio, cFGF23, and iFGF23 significantly increased with no between‐group differences. Klotho was unchanged. 1,25(OH)_2_D and PINP significantly increased in the 24,000 and 48,000 IU groups. SOST, OPG, RANKL, BMD, BMC, and CTX remained unchanged. In subgroup analyses restricted to participants deficient (25(OH)D <25 nmol/L) at baseline, findings were similar. There were no significant changes in BMD, BMC, and CTX. Although an increase in PINP was not found in this subgroup, the PINP/CTX ratio increased.

Before supplementation, plasma concentrations of both total and free 25(OH)D were associated with cFGF23 and PTH but not any of the markers of Wnt signaling or bone metabolism, except for BAP. Both free and total 25(OH)D were positively associated with BMD and BMC of both sites at baseline. After supplementation, total and free 25(OH)D was positively associated with DBP (*p* < 0.001) and negatively with adjusted calcium and eGFR (*p* < 0.01). The negative association with PTH and positive association with SOST (*p* < 0.05) were only significant for free 25(OH)D after supplementation. There were no significant associations with other markers of Wnt signaling and bone metabolism. The relationships with BMD and BMC were no longer found after supplementation.

The expected dose‐dependent increase in total and free 25(OH)D and 24,25(OH)_2_D with vitamin D supplementation was observed in this study. This was accompanied by a dose‐dependent decrease in PTH, as observed before in generally heathy people.^(^ ^37^, ^38^, ^39^, ^40^, ^41^ ^)^ We also found an increase in 1,25(OH)_2_D concentrations in the 24,000 and 48,000 IU/mo groups, despite the fact that few of the study participants had baseline values of 25(OH)D below the concentration usually considered as rate limiting for 1,25(OH)_2_D production. This was also observed in other studies.^(^ ^42^ ^)^

We found a significant increase in cFGF23 and iFGF23 with vitamin D supplementation, some individuals exceeding the normal ranges of cFGF23 and iFGF23 (laboratory‐specific normal range: cFGF23 <100RU/mL^(^ ^43^ ^)^; iFGF23 28–121 pg/mL as established in 50 healthy individuals; personal communication Professor WD Fraser). An increase in iFGF23 with vitamin D supplementation was also reported in a recent meta‐analysis.^(^ ^44^ ^)^ This may be partly mediated by the increase in 1,25(OH)_2_D observed in our study. There is a reciprocal regulation of FGF23 and 1,25(OH)_2_D^(^ ^42^ ^)^; 1,25(OH)_2_D stimulates the expression of FGF23 and Klotho^(^ ^42^, ^45^, ^46^, ^47^ ^)^ and in reverse, Klotho has been shown to stimulate 25(OH)D activation in the kidney.^(^ ^18^ ^)^ FGF23, however, stimulates the expression of CYP24A1, thereby increasing catabolism of 1,25(OH)_2_D and the conversion of 25(OH)D into 24,25(OH)_2_D,^(^ ^17^, ^48^ ^)^ while at the same time inhibiting CYP27B1 expression and thus 1,25(OH)_2_D production.^(^ ^42^ ^)^ FGF23 has also been reported to inhibit PTH synthesis production, a mechanism modulated by 1,25(OH)_2_D.^(^ ^42^ ^)^

The increase in FGF23 may be secondary to an increase in intestinal calcium and phosphate absorption^(^ ^49^ ^)^ as mediated by the increase in 1,25(OH)_2_D. It may thus reflect a compensatory response to maintain phosphate homeostasis by increasing FGF23‐mediated urinary phosphate excretion.^(^ ^17^, ^42^, ^50^ ^)^ Accordingly, we found a decrease in plasma phosphate after supplementation. Also, PTH has a phosphaturic effect; therefore, the increase in FGF23 may also be a response to the observed decrease in PTH in this study.

Elevated plasma concentrations of cFGF23 and iFGF23 are found from early stages of renal impairment followed by an increase in plasma phosphate and PTH as CKD progresses.^(^ ^42^, ^51^ ^)^ This is associated with soft tissue calcification and increased risk of cardiovascular disease (CVD).^(^ ^52^ ^)^ It is also associated with low plasma concentrations of 1,25(OH)_2_D due to the aforementioned FGF23‐mediated inhibition of production and increased catabolism. It may therefore contribute to the risk of osteomalacia and negative association with markers of bone integrity and fracture risks, particularly in trabecular bone.^(^ ^53^, ^54^, ^55^ ^)^ Whether an increase in FGF23 in response to vitamin D supplementation without a concomitant increase in plasma PTH and phosphate and decrease in 1,25(OH)_2_D is also associated with negative bone and CVD health outcomes needs further investigation.

Renal function is an important determinant of the response to vitamin D supplementation.^(^ ^41^ ^)^ In post‐supplementation ANCOVA models that included eGFR as a covariate, eGFR was significant for total and free 25(OH)D and cFGF23. The interaction of eGFR with post‐supplementation 25(OH)D may reflect increased catabolism and impaired dose‐response associated with a decline in renal function.^(^ ^56^ ^)^ The interaction with cFGF23 may also be explained by the importance of renal function in the catabolism and urinary excretion of FGF23 fragments.^(^ ^19^ ^)^ In models for markers of Wnt signaling, bone metabolism, and BMD and BMC, eGFR was not significant. It is possible that the effect of renal function on bone markers may predominantly be observed at a lower eGFR than observed in this cohort.

Our study did not confirm an anabolic effect of vitamin D supplementation on components of the Wnt signaling pathway.^(^ ^26^, ^27^ ^)^ The regulators SOST, OPG, and sRANKL remained unchanged, whereas DKK1 significantly increased in two groups. Data on the effect of vitamin D supplementation on RANKL and OPG are conflicting.^(^ ^57^ ^)^ Some studies reported that 1,25(OH)_2_D can decrease the expression of RANKL and upregulate OPG/RANKL. This is partly mediated through the inhibitory effect of 1,25(OH)_2_D on inflammatory factors.^(^ ^58^, ^59^ ^)^ However, another study suggests that 1,25(OH)_2_D increases the expression of RANKL and decreased OPG and enhanced osteoclast formation.^(^ ^60^ ^)^ Although no pronounced effects on these regulators of bone metabolism were found, there was an increase in the formation marker PINP in the two highest‐dose groups, while the PINP/CTX ratio increased in all groups. This may indicate that the balance of bone formation and resorption may have changed with supplementation, consistent with other studies.^(^ ^61^, ^62^ ^)^

Vitamin D supplementation may increase bone mineralization^(^ ^63^ ^)^ and therefore BMD and BMC by increasing the bioavailability of calcium and phosphate.^(^ ^64^ ^)^ This may be independent of potential effects of increased vitamin D status on alterations of bone cell differentiation and function. This is most pronounced when substantial amounts of unmineralized bone matrix are present before supplementation commences, such as with osteomalacia, associated with a plasma concentration of 25(OH)D below 25 nmol/L.^(^ ^24^, ^29^ ^)^ Therefore, the effects of supplementation may have depended on vitamin D deficiency at baseline. In the VDOP study, 28% of participants had a baseline of 25(OH)D <25 nmol/L. This study and earlier analyses of the VDOP trial showed no interaction between the presence or absence of baseline vitamin D deficiency and change in BMD of the hip and femoral neck,^(^ ^28^ ^)^ markers of Wnt signaling and bone metabolism, except for PINP. However, our study was not powered for this subgroup analyses.

Regression analyses showed a positive association of BMD and BMC and both total and free 25(OH)D at baseline. This is consistent with other cross‐sectional studies.^(^ ^29^ ^)^ The lack of an effect of supplementation appears to be contradictionary to these findings. However, in unsupplemented individuals, 25(OH)D likely reflects a wider range of factors influencing both vitamin D status and BMD and BMC, such as time spent outdoors, physical activity, or body composition.^(^ ^25^, ^65^ ^)^ The associations between 25(OH)D and BMD and BMC were no longer significant after supplementation. This might indicate that after supplementation, 25(OH)D concentration ranges were achieved within which a further increase does not result in an increase in mineralization. In addition, after supplementation, vitamin D status will predominantly have been determined by oral intake and as such may override the effect of before‐mentioned lifestyle factors on 25(OH)D. Surprisingly, both at baseline and post‐supplementation, no significant associations were found with any of the measured markers of the Wnt signaling pathway or bone metabolism, except for BAP.

It has been suggested that serum‐free 25(OH)D may be a better measure of tissue availability and utilization and may be a better predictor of functionality of vitamin D than total plasma 25(OH)D.^(^ ^66^, ^67^ ^)^ The majority of vitamin D metabolites circulate bound to DBP (85% to 90%) or albumin (10% to 15%) and only a small fraction circulates in its free form.^(^ ^66^, ^67^ ^)^ According to the free hormone theory, only the free fraction can enter cells, unless the megalin/cubilin‐mediated endocytotic uptake allows for internalization of DBP‐bound metabolites. This has so far only been demonstrated in the kidney, breast, and muscle tissue.^(^ ^66^ ^)^ Bone cells may therefore depend on the free 25(OH)D concentration in their microenvironment, where local conversion to 1,25(OH)_2_D takes place^(^ ^68^, ^69^ ^)^ with auto and paracrine effects. In healthy individuals, total and free 25(OH)D are highly correlated^(^ ^66^, ^67^, ^70^ ^)^ and generally have the same relationships with health outcomes.^(^ ^57^, ^66^, ^67^ ^)^ This was also found in this study. The concentration of DBP itself may be a determinant of the concentration of vitamin D metabolites because it protects against catabolism, thereby prolonging half‐life.^(^ ^71^ ^)^ The significant association between DBP and plasma 25(OH)D in this study appears to confirm this.

This study has several limitations. The absence of a placebo group did not allow to account for changes unrelated to the intervention (ie, effect of aging or secular trends). Our study was not powered for subgroup analyses by baseline vitamin D status. The length of supplementation may have been too short to detect significant changes in BMD and BMC as measured by DXA. We, however, also did not observe the anticipated 0.6% decrease in BMD, the average annual change in BMD in this age group,^29^, ^30^ ^)^ the study was powered to detect.^(^ ^28^ ^)^ Markers of bone metabolism and osteocyte signaling may, however, be expected to respond to interventions more rapidly and within the length of a bone remodeling cycle (~3 to 4 months).^(^ ^72^ ^)^ It is possible that markers measured after 12 months reflect a newly achieved steady state that is seemingly no different from baseline and that changes occurred within the first few months after commencement of the intervention, such as observed in pharmaceutical trials.^(^ ^70^, ^72^ ^)^ We did not directly measure free 25(OH)D but instead calculated the free fraction; these two approaches may have provided different findings.

In conclusion, the decrease in PTH and increase in PINP/CTX ratio suggest a protective effect of supplementation on bone metabolism, although no significant effect on BMD or pronounced changes in regulators of the Wnt signaling pathway were found. Also, no changes in BMD were found in subgroup analyses restricted to participants who were vitamin D deficient at baseline. The increase in FGF23 warrants caution due to its negative associations with bone and cardiovascular health. Relationships between total and free 25(OH)D concentrations with biomarkers were similar and confirmed positive associations of higher vitamin D status and BMD. The change in associations after supplementation might suggest a threshold effect.

## Disclosures

MC, IP, CW, and JCYT have no conflicts of interest to disclose. TJA has served on an advisory board and received lecture fees from Internis. WDF has received research grants, sat on advisory boards, and given lectures on behalf of Eli Lilly, NPS Pharmaceuticals, Shirel, Entera Bio Ltd., and Nycomed. IS has served on advisory boards.

## Author Contributions

**Marilena Christodoulou:** Conceptualization; formal analysis; methodology; project administration; visualization; writing – original draft; writing – review and editing. **Terence J Aspray:** Conceptualization; data curation; funding acquisition; investigation; resources; supervision; validation; visualization; writing – original draft; writing – review and editing. **Isabelle Piec:** Investigation. **Christopher Washbourne:** Investigation. **Jonathan CY Tang:** Investigation. **William D Fraser:** Resources; writing – review and editing. **Inez Schoenmakers:** Conceptualization; data curation; formal analysis; funding acquisition; investigation; methodology; project administration; resources; supervision; validation; visualization; writing – original draft; writing – review and editing.

### Peer Review

The peer review history for this article is available at https://publons.com/publon/10.1002/jbm4.10619.

## Data Availability

Primary data and outcomes were published per EUDRACT protocol.
